# Providing Accessible Recreation Outdoors—User-Driven Research on Standards (PARCOURS): Protocol for a Multiphase Study

**DOI:** 10.2196/33611

**Published:** 2022-03-31

**Authors:** Mike Prescott, François Routhier, Delphine Labbé, Marie Grandisson, Atiya Mahmood, Ernesto Morales, Krista L Best, Mir Abolfazl Mostafavi, Jaimie Borisoff, Stéphanie Gamache, Bonita Sawatzky, William C Miller, Laura Yvonne Bulk, Julie M Robillard, Hailey-Thomas Jenkins, Kishore Seetharaman, W Ben Mortenson

**Affiliations:** 1 Center for Interdisciplinary Research in Rehabilitation and Social Integration Centre intégré universitaire de santé et de services sociaux de la Capitale-Nationale Québec City, QC Canada; 2 Centre for Research in Geospatial Data and Intelligence Université Laval Québec City, QC Canada; 3 Department of Rehabilitation Université Laval Québec City, QC Canada; 4 Disability and Human Development Department University of Illinois Chicago, IL United States; 5 Department of Gerontology Simon Fraser University Vancouver, BC Canada; 6 Department of Occupational Science & Occupational Therapy University of British Columbia Vancouver, BC Canada; 7 Department of Geomatics Sciences Université Laval Québec City, QC Canada; 8 Rehabilitation Engineering Design Lab British Columbia Institute of Technology Burnaby, BC Canada; 9 International Collaboration on Repair Discoveries Vancouver, BC Canada; 10 Department of Orthopedics University of British Columbia Vancouver, BC Canada; 11 GF Strong Rehabilitation Research Program Vancouver, BC Canada; 12 Department of Medicine Division of Neurology University of British Columbia Vancouver, BC Canada

**Keywords:** parks, accessibility, standards, user-oriented research

## Abstract

**Background:**

Canada’s national parks are world-renowned. However, despite recent attempts to improve access, many are not accessible to people with disabilities. With the advent of provincial and federal legislation, standards are being developed to assist with the design and management of parks.

**Objective:**

The overarching objective of this study is to inform accessibility standards for federal parks that meet the needs of all park visitors, regardless of ability. The specific objectives of this study are to identify park accessibility standards that exist internationally, identify the accessibility challenges that people with disabilities face in park environments, and prioritize and recommend accessibility standards for national parks.

**Methods:**

A 3-phase approach will be used to achieve the study objectives. In the first phase, a scoping review of the existing accessibility standards will be conducted. The second phase will include objective audits of trails and features in 6 parks, 3 in western Canada and 3 in eastern Canada, as well as mobile interviews with 24 diverse participants in each region regarding their experiences of and recommendations for improving the park’s accessibility. In the final phase, a Delphi participatory consensus development process will be used, based on the data gathered in the first 2 phases, to prioritize recommendations for standards.

**Results:**

We expect to find gaps in existing standards that do not account for the diverse range of accessibility requirements that people with disabilities have for visiting parks. We also expect to find that existing standards, on their own, may not be enough to ensure equitable access to all the experiences and amenities that parks have to offer. Development of subsequent guidelines and best practices may be necessary to address complex scenarios for which standards may not be the best approach to ensuring accessibility.

**Conclusions:**

The participatory and mixed methods approaches used in this study will provide rich insights for developing accessible park standards that consider the diverse needs of people with disabilities. The findings will also support the development or enhancement of park standards at all levels of government.

**International Registered Report Identifier (IRRID):**

DERR1-10.2196/33611

## Introduction

### Background

Outdoor natural parks offer a variety of experiences that result in physical and psychological [1-3] as well as social and health benefits of access to green and blue spaces [3-10]. However, people with disabilities, who represent 22.3% of the population in Canada [11] and 25.7% in the United States [12], are often excluded from these spaces because of accessibility issues [13]. Limited access to outdoor spaces further contributes to the inequities that people with disabilities already face in employment, housing, and health care [14]. In response to challenges in built and natural environments, the Canadian federal government has enacted a legislation called the Accessible Canada Act to remove barriers to participation for people with disabilities [15]. This legislation includes a road map for developing accessibility standards that regulate organizations under federal responsibility, such as national parks. The intent is that these national standards will be adopted at all levels of government so that people with disabilities can expect the same level of service from every park they visit.

Historically, standards have focused on promoting access for people with physical disabilities, often neglecting the needs of people who experience cognitive, sensory, or multiple disabilities [16]. For example, wayfinding is emerging as a critical topic for different disabilities to identify accessible routes for planning purposes and to enable real-time navigation. Specific wayfinding standards also need to consider the dynamic nature of the environment to foster accessibility during trail construction, snowmelt, or massive rainstorms. In these instances, it is important that information about alternate routes consider accessibility requirements. The challenge of developing standards is compounded by the variety of assistive devices used by and capacities of people with diverse disabilities. Currently, some people with disabilities are excluded because the size of their mobility devices exceeds the space provided under existing building codes [17]. The use of hand cycles (3- or 4-wheeled cycles propelled by the arms rather than the legs) in parks will also require standards that mandate wider paths for turning [18]. Further complications arise when designs that meet the needs of one group conflict with the needs of others. For example, tactile paving at crosswalks, which warns those with vision loss that they are entering a street, can be hazardous for manual wheelchair users who may find that it is uncomfortable or causes pain [19] or might precipitate a fall [20]. Therefore, it is important to develop standards that consider and involve people with a wide variety of disabilities in the process.

**Figure 1:**
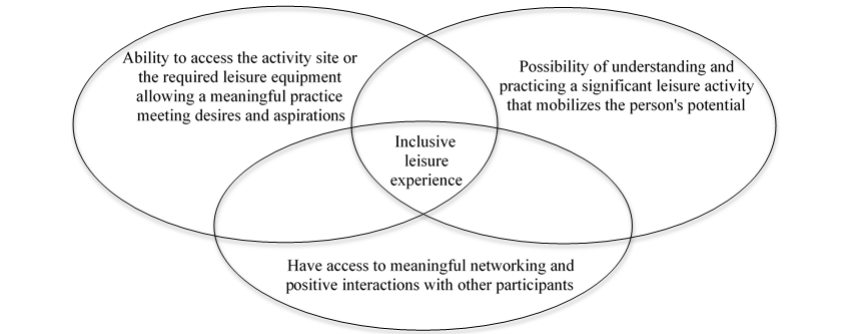
Components of an inclusive leisure experience [22].

Carbonneau et al [21] developed a conceptual framework (Figure 1) that promotes inclusive leisure experiences for people with disabilities in their communities. Being present and participating in some aspect or aspects of an activity does not guarantee the quality of the experience [21]. Not only do we need to consider the physical components of access, but we also have to take into consideration the significance of the activity for the participant and the necessity of positive interactions with other participants. Thus, if we wish to inform standards to make parks more inclusive, the experiences and preferences of people with disabilities need to be understood.

### Objectives

The overarching purpose of this participatory project is to inform the development of standards to make parks more accessible. The acronym for the project entitled Providing Accessible Recreation Outdoors: User-driven Research on Standards is PARCOURS. This is a French word for *trail*, that is, *un chemin pour aller d'un point à un autre*, which emphasizes our project’s bilingual focus on developing standards to improve accessibility in parks across Canada. The specific objectives of this study are as follows: (1) to identify park accessibility standards that exist internationally; (2) to identify the accessibility challenges that people with disabilities face in park environments; and (3) to prioritize and recommend accessibility standards for national parks.

## Methods

### Overview

The research will be conducted in 3 phases over a 24-month period and in two provinces: British Columbia and Québec. The methods described in this proposal will be the same in both provinces. Advisory committees including individuals with a variety of disabilities have been created in both provinces (one in each province) to ensure the consideration of inputs or concerns of these individuals in the research project through a participatory research approach. These committees include individuals with mobility, visual, and hearing disabilities; intellectual disabilities; autism spectrum disorder; dementia; and Alzheimer disease. The committees will meet 2 to 3 times a year to provide feedback on the ongoing phases of the research project that are presented below. They will help us fine-tune the protocol.

A scoping review of existing standards will be conducted in the *first phase*. In the *second phase*, park audits and mobile interviews will be conducted with people who have a wide range of disabilities at 3 parks in each province in the summer and winter. The *third phase* will use the data collected in the first 2 phases to inform the selection and prioritization of park standards using a Delphi process. A final report will be presented to the granting agency (Accessibility Standards Canada) at the end of the study. The study has received approvals by research ethics boards in both provinces.

### Phase 1: Scoping Review

The objective of the scoping review will be to compare and contrast existing international and national standards, along with novel research evidence to inform the development of revised standards. The scoping review will involve five steps: (1) identifying the research question, (2) identifying the key words, (3) identifying relevant standards and guidelines, (4) choosing standards, and (5) charting the data and reporting the results [22]. The question guiding the search for relevant studies will be, “What are the current accessibility standards in terms of outdoor spaces, including parks to allow people with disabilities to enjoy the natural environments?” The scoping review was conducted between June 2020 and February 2021 using Google search and governmental or official park websites. The search keywords will include accessibility terms (eg, access* standard*, disabilit* policy, regulation*, and guidelines), parks and nature (eg, parks, outdoors, natural, urban, trail*, path*, and national), mobility device, and disability types (eg, wheelchair*, scooter*, blind, partial sight, deaf, hard of hearing, cognitive, mental, and developmental). The search will cover international (eg, United States, World, World Health Organization, Europe, France, Switzerland, United Kingdom, England, Australia, and Spain) and Canadian national standards, including provincial guidelines. For feasibility purposes, we will exclude the guidelines and standards from the municipal level. The search will be conducted both in English and French, and some standards in Spanish will also be included, as these 3 languages are spoken by the research team. The data will be extracted and charted based on the features listed on the Parks Canada website and completed with the content of the other standards found. This list of features included paths and trails (eg, sidewalks, walkways, stairs, ramps, lighting, and obstacles), parking and drop-off areas and transit areas, amenities (eg, rest areas, visitor centers, outdoor shelters, point-of-sales, and washrooms), wayfinding and signage, park management (eg, policies, practices, and communications), and summer and winter activities (eg, access to activities, equipment, and installations). This research will provide an overview and critique of the existing standards on outdoor spaces, along with the possible knowledge gaps on the subject.

### Phase 2: Park Audits and Mobile Interviews

In phase 2, physical audits of accessibility will be conducted on-site, and people with disabilities will walk or wheel along a portion of these routes. The focus of the audits and participant interviews will be on park trails and features along the trails.

#### Park Audits

Approximately 10 km of park trails in 3 parks in each province will be audited for their accessibility, including the trails participants will be used in the mobile interviews. This will be used to provide context for the analysis of the conditions faced by participants in the mobile interview. Trail slope, cross slope, width, surface quality (ie, firm, level, and stable), and presence of obstacles and hazards will be measured and mapped using the High Efficiency Trail Assessment Process (HETAP; Beneficial Designs) cart equipped with automated GPS, distance and slope sensors, and a camera. Parks will be chosen to include a variety of settings (ie, mountains, coastlines, and forests) and features (eg, beaches, picnic areas, and camping) found in most national parks. The closest national or subnational parks (ie, provincial and regional) that have a diversity of features will be used to minimize travel for participants. This process will allow us to objectively assess the parks for their specific characteristics and provide a portrait of possible obstacles users can face while visiting parks.

#### Mobile Interviews

##### Overview

Mobile interviews will be conducted in three steps: a preinterview survey, a mobile interview, and study-specific interview activities. These mobile interviews were performed in parks with users living with various disabilities to gather more information on the lived experience of individuals and how, according to their abilities, their park experience is influenced. Mobile interviews allow the identification of unforeseen sociospatial interactions compared with traditional face-to-face interviews [23] and can help people with disabilities think about elements that they would not think of if they were not directly in the environment [24]. Participatory research is a broad method that can be carried out in many ways, depending on the objectives. The mobile interview method used is one way to accomplish our objective. As specified at the beginning of the Methods section, we have advisory committees that include individuals with lived experience and disability organization leaders to guide us through the process. The mobile interview process described in step 2 specifies how we are going to involve individuals in the process.

##### Step 1 (Preinterview Survey)

Each participant will complete a web-based questionnaire (Qualtrics) that asks about sociodemographic characteristics (eg, age and sex), disability and mobility status (eg, diagnoses and assistive aids used), wayfinding skills, preferences for park settings and activities, and transport mode to parks. A few days before the interview, participants will be contacted to remind them of the interview, review survey responses, and review the assigned park website to evaluate whether or not it provides the information they would need to feel confident about visiting the park.

##### Step 2 (Mobile Interview)

Interviews will take place in the park assigned to the participant [25-27]. The interviews will be administered by trained researchers. They will assist participants where necessary and ensure health precautions are followed. The mobile interview will take approximately 2 hours along 3 predetermined routes of 500-1300 m in length [26]. Participants will be encouraged to take breaks as needed.

Before starting a route, participants will be given a map of the intended route to help orient them and asked what their expectations are for the route (eg, how far do they think the route is, how hard will it be, and do they think they will enjoy it?). Specific adaptations will be made to the map for individuals with vision loss (ie, describe the map). The map will include the trail that will be used, features along the route, and landmarks that might assist their travel. Researchers will retrieve the map but make it available during the journey when requested. This will be repeated before each route. While traveling through the park, researchers will use a GoPro 8 (GoPro Inc; with GPS device) to film the participants and an additional audio recorder to capture the discussion along the journey. Researchers will follow participants as they travel along the route, redirecting them to the prescribed route if necessary. During this process, researchers will ask structured and semistructured questions about their experiences and take notes of any additional observations they see (eg, the participant appears to be struggling with the terrain and the entire width of the path was covered in mud). The semistructured component of the interview guide (Textbox 1) will focus on participants’ experiences related to wayfinding and wayfaring.

**Semistructured interview questions about wayfinding and wayfaring experiences.**

**Wayfinding questions**

- What direction do you think we should go? Why do you think this?
- Can you show on the map where we are and where we are going?
- What cues are you using to make that decision and why?
- What is drawing your eye or attention?

**Wayfaring questions**

- Routes
  How does this path feel to you?
  How safe do you feel along this route? Why?
  How comfortable is this path for you? Why do you feel this way?
  How do you feel about the overall experience of this path?
  Are there any changes you would like to see made to this path?
- Features (eg, bench, picnic table, and washroom)
  What are your thoughts about using this feature?
  How would you use this feature?
  Are there any changes you would make to this feature?

Structured questions about the presence or absence of features (eg, public toilet) and their characteristics (eg, accessibility and maintenance) will be asked as concise readable statements (eg, “The path leading to the public toilet is accessible”). This component draws on a park-specific adaptation of the Stakeholders Walkability/Wheelability Audit in Neighbourhood (SWAN) tool, a user-led microscale environmental audit tool that captures both objective and subjective data identifying features that hinder or support mobility and participation of people with mobility disabilities across five domains (ie, functionality, safety, land use features or destinations, maintenance or esthetics, and social aspects) [28]. Pilot testing of the original tool showed good interrater agreement across 90% of the items on the tool [29]. The park-specific adaptation is called the Stakeholders Walkability/Wheelability Audit in Nature (SWAN-Parks) tool. It includes blocks of questions associated with the aforementioned five domains that are tailored to different areas in the park, namely, amenities (eg, washrooms), paths (ie, that connect between different amenities), and trails. These blocks of questions were developed based on extant empirical evidence on accessibility issues in parks for different groups of people with disabilities, as well as pre-existing validated park audit tools, such as the Community Stakeholder Park Audit Tool [30] and Natural Environment Scoring Tool [31]. The semistructured and structured questions are sequenced into a cohesive interview guide and customized against the preidentified interview routes. Research assistants will orient participants (and personal assistants) to the different types of questions being asked during the mobile interview and provide prompts and clarification on an ongoing basis as they move through the mobile interview.

Sighted participants will also be asked to wear glasses that help track their gaze (Tobii Pro Glasses; 50-Hz eye-tracking glasses). These glasses will track their gaze during the journey to understand where they focus their attention during travel, including hazards, enjoyable features, and landmarks.

##### Step 3 (Postroute Interview Questions)

**Figure 2:**
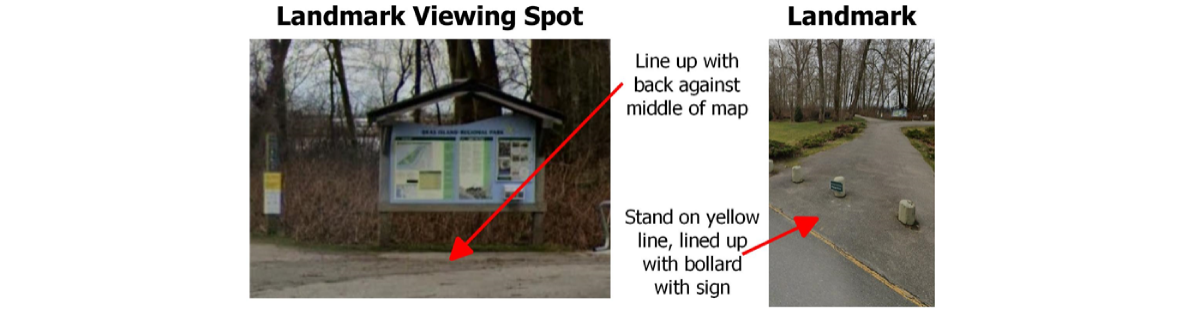
Test of objective spatial skills.

At the end of each route, participants will be asked to complete a series of tasks. First, participants will rate the route on a 7-point Likert scale, their perceived physical demand, mental demand, safety, enjoyment, and confidence to find their way independently. Follow-up questions for each scale would be regarding which changes they would recommend, including providing equipment or changes to the environment. Participants will also be asked to recall the route verbally or by drawing the route and all its features onto a route map [32]. The participant will be asked to describe the wayfaring and wayfinding experiences overall and provide additional feedback and recommendations. The final task will be to test objective spatial skills, including orientation and estimation skills necessary to the park environments [33]. Participants will be positioned at a predefined location and asked to point a compass in the direction of the origin of the route to measure orientation skills. At this same location, they will also be asked to estimate the distance and slope to a predefined landmark in the distance (Figure 2) [34,35]. Orientation and estimation skills are essential for reaching destinations and learning routes for future travel [36]. These skills may enhance confidence and encourage greater use of parks [37]. This information may also help parks to identify signage needs, including the type of information needed on those signs (eg, route slopes and distance markers). For winter evaluations, the data collection process will be repeated, changes in accessibility due to seasonality will be noted.

#### Recruitment

A purposive sample of 48 people (24 at each site) with a broad range of disabilities who use a variety of mobility devices will be eligible to participate in this study. To be included, participants will be at least aged 18 years, able to travel approximately 3 km with rests over a 2- to 3-hour period, and able to communicate directly with researchers (verbally) or indirectly through an assistant or attendant (Table 1 describes the sample distribution according to their individual characteristics).

Participants will be recruited through partners and participants from previous studies and selective advertising, if necessary. As there are 8 disability groups, 24 participants, and only 3 parks, participants will be assigned, where possible, to a park to ensure maximum variation in each park. Each participant will visit 1 of 3 park sites for a walking or wheeling interview during the summer months. Half of those participants (12/24, 50%) will visit the same park site in winter to account for the impacts of seasonal variation on potential accessibility standards. Participants in each province will complete 36 interviews, resulting in 72 separate interviews.

**Table 1:** Sample distribution according to participant characteristics (n=24).

Characteristics	Participants, n (%)
Scooter users	3 (13)
Power wheelchair users	3 (13)
Manual wheelchair users	3 (13)
Walker users	3 (13)
Cane or crutches users	3 (13)
People with visual impairments (including both white cane users and guide dog users)	3 (13)
People with hearing impairments	2 (8)
People with cognitive impairment (eg, dementia, autism, and intellectual disabilities)	4 (17)

#### Data Analysis

##### Participant Characteristics

Survey responses from step 1 and objective spatial skills tests from step 2 will be used to summarize the characteristics of the sample. Descriptive analysis will include counts of nominal data, means, and SDs for participant’s socioeconomic status, mobility, assistive device use, and wayfinding skills. Results of the spatial skills tests will be reported as absolute error (pointing error in degrees, distance estimation in meters, and slope estimation in percent) and as relative error in percent.

##### Descriptive Analysis

The descriptive analysis will be carried out as follows:

- Concerning interview results, video and audio from the mobile interview will be transcribed to indicate what was being said or observed and by whom. Each quotation will be coded to reflect the feature or experience being explained (wayfinding or wayfaring) by the participant and any observation made by the researchers. The quotation and its code will be digitized in the geographic information system (GIS) at the location where it occurred. This will be linked to the participant survey responses through their ID as a separate file in the GIS (delimited file without spatial information). The results from the SWAN, the accessibility audit in phase 1, and sketch maps from the interview will also be uploaded into the GIS. They will be used to provide context about the physical environment and participants’ familiarity and recommendations. These layers can then allow for filters to be used during the analysis based on any data collected during the survey. Wayfinding results include feedback from participants during the interview and researcher observations about wayfinding behaviors.
- A summary of the gaze position of participants will be completed by categorizing the amount of time looking at specific items and general gazing locations (eg, do manual wheelchair and walker users spend more time looking down at the ground compared with power wheelchair users or those who are able to see and walk?). Eye gaze data (analyzed with iMotions Eye Tracking Module software) will be added as a layer in the GIS that will be reviewed to determine if patterns exist between layers (topographical, spatial transcript, sketch maps, video evidence, and researcher observations). These findings will be compared between mobility groups and between environments to determine if there are differences in how individuals with disabilities experience the environment and use landmarks to recall routes.

##### Global Analyses

The scoping review provides us with an additional layer of information that we can use to compare with the HETAP objective trail data and the subjective information from the mobile interviews. We can compare the subjective and objective layers using the existing standards from the scoping review.

### Phase 3: Modified Delphi for the Prioritization and Identification of Standards

The Delphi panel process (participatory consensus developing process) [38,39] will be used to establish consensus and prioritize recommendations for accessibility standards using several national panels that focus on specific park areas (eg, trails and paths, information, and services). Drawing on what we will learn during the systematic review conducted in phase 1, and from the mobile interviews conducted in phase 2, we will then conduct additional individual interviews and focus groups of approximately 40 participants (including people with a wide spectrum of physical, cognitive, and sensory disabilities, as well as accessibility experts). Through a semistructured interview of approximately 1 hour, these experts will be asked to share their opinion on the hierarchy on which accessibility standards should be considered. The standards will be presented, and other participants’ opinions will be shared, and the participants will have to comment.

During the second and third rounds of the Delphi, we will use a multimodal and iterative approach to encourage participants to reflect on existing standards and make recommendations for new standards. This process will include up to 100 participants (English- and French-speaking individuals from a range of ages, cultures, ethnicities, and income levels) that can participate with the assistance of a research assistant or independently on the web. Depending on the number of potential standards, these participants may be subdivided so they can review potential standards that are categorized into more manageable subgroups (eg, 3 or 4). From this process, we will create a list of recommended accessibility standards. The use of the Delphi process as it is presented considers the fact that we have 2 sites (French- and English-speaking) that we both want to consider rather than considering 2 separate sites. It also allows us to reach out to a greater number of experts in various fields.

### Ethics

The protocol for this study was approved by the Research Ethics Boards at the University of British Columbia (H20-04036), the Centre intégré universitaire de santé et de services sociaux de la Capitale-Nationale (Project 2021-2120), and regional health authorities at each site. All study participants will provide informed consent. Evaluation in parks began in May 2021.

## Results

Funding for this study was obtained from the Accessibility Standards, Canada. Results from the study will be reported for all phases including the systematic review, park audits, mobile interviews, and the Delphi process. A variety of spatial transcripts will be developed that show patterns in the data according to personal characteristics, age ranges, gender, or assistive device use or disability. A report of the final recommendations will conclude the results.

## Discussion

### Principal Findings

The purpose of this protocol is to describe the methodology for informing park accessibility standards. The combination of data gathered on parks and their use by individuals with various disabilities as well as the participatory approaches used to discuss existing standards should allow a better understanding of the conditions and dynamics required to propose positive inclusive leisure experiences in parks.

The data collected will inform future national park accessibility standards. Having a repository of existing standards (phase 1) and evidence supporting them will be beneficial to others who are undertaking the development of similar standards, to other levels of government in Canada, and internationally. It is anticipated that others will adopt this methodology to create user-driven accessibility standards that will have the following characteristics: (1) promote the widespread inclusion of people with disabilities in these spaces; (2) introduce broadly applicable standards (rather than siloed) and promote the widespread inclusion of people with disabilities in these spaces; (3) facilitate access by their families, parents with children, and older adults who may not self-identify as having a disability; (4) make park managers aware of the accessibility information that people with disabilities and their families need to plan trips to parks and enjoy their visits; (5) assist decision-makers in assessing to make improvements to parks and that people with disabilities and their families will use that information to make decisions about their visits; (6) continually be reviewed and assessed to ensure they meet the needs of people with disabilities as technologies and demand evolve; (7) create demand for similar mapping exercises to be undertaken in other parks; (8) identify modifications required for existing standards and new standards that should be developed; and (9) be adopted federally and that they will inform the development of similar standards provincially and municipally.

### Limitations

The nature of the challenge addressed in this study introduced some limitations. The sample size was based on having adequate representation of all groups in different settings similar to federal parks but was fairly small. Given that the data will be collected outdoors, we were restricted by the nature of the exercise and requirements of the activity. Taking into consideration the grant funding and the fact that we wanted to offer a generous stipend to the participants, plus the cost of the equipment used, we were also limited in the number of participants we were able to reach. Moreover, the COVID-19 pandemic forced us to increase researcher and participant safety measures by using parks that were not at the national level because they were closer to the participants’ homes and required less travel. This may result in not assessing every feature or experience that might be expected in a national park. Limitations due to the size of our sample likely mean that not every potential perspective needed to inform standards could be accounted for in a population of such diversity of abilities, assistive device use, and park preferences. Finally, we may be limited in the causal conclusions that may be drawn because the study was not experimental in nature. This may be offset somewhat using multiple methods (eg, systematic review, mobile interview, and the Delphi method), the composition of the research team, and guidance from partners and the advisory committee.

### Conclusions

This study will provide valuable insights from people with disabilities for recommending accessibility standards to be used in national parks and beyond. Although standards are not the only part of an effective inclusive park strategy, they are necessary for establishing a common language and set of expectations for accessibility in these spaces. They establish a baseline for park agencies to build on and ensure that the tremendous benefits they provide are available to all.

## Abbreviations

GIS: geographic information system
HETAP: High Efficiency Trail Assessment Process
SWAN: Stakeholders Walkability/Wheelability Audit in Neighbourhood

## References

[1] Jakubec S, Carruthers Den Hoed D, Ray H, Krishnamurthy A (2016). Mental well-being and quality-of-life benefits of inclusion in nature for adults with disabilities and their caregivers. Landscape Res.

[2] Saitta M, Devan H, Boland P, Perry MA (2019). Park-based physical activity interventions for persons with disabilities: a mixed-methods systematic review. Disabil Health J.

[3] Gascon M, Zijlema W, Vert C, White MP, Nieuwenhuijsen MJ (2017). Outdoor blue spaces, human health and well-being: a systematic review of quantitative studies. Int J Hyg Environ Health.

[4] James L, Shing J, Mortenson WB, Mattie J, Borisoff J (2018). Experiences with and perceptions of an adaptive hiking program. Disabil Rehabil.

[5] Labbé D, Bahen M, Hanna C, Borisoff J, Mattie J, Mortenson WB (2019). Setting the sails: stakeholders perceptions of an adapted sailing program. Leisure Sci.

[6] Markevych I, Schoierer J, Hartig T, Chudnovsky A, Hystad P, Dzhambov AM, de Vries S, Triguero-Mas M, Brauer M, Nieuwenhuijsen MJ, Lupp G, Richardson EA, Astell-Burt T, Dimitrova D, Feng X, Sadeh M, Standl M, Heinrich J, Fuertes E (2017). Exploring pathways linking greenspace to health: theoretical and methodological guidance. Environ Res.

[7] Merrick D, Hillman K, Wilson A, Labbé D, Thompson A, Mortenson WB (2021). All aboard: users' experiences of adapted paddling programs. Disabil Rehabil.

[8] Rugel E Green space and mental health: pathways, impacts, and gaps. National Collaborating Centre for Environmental Health.

[9] Rugel EJ, Carpiano RM, Henderson SB, Brauer M (2019). Exposure to natural space, sense of community belonging, and adverse mental health outcomes across an urban region. Environ Res.

[10] Shanahan DF, Bush R, Gaston KJ, Lin BB, Dean J, Barber E, Fuller RA (2016). Health benefits from nature experiences depend on dose. Sci Rep.

[11] Morris S, Fawcett G, Brisebois L, Hughes J A demographic, employment and income profile of Canadians with disabilities aged 15 years and over, 2017. Canadian Survey on Disability Reports.

[12] Okoro CA, Hollis ND, Cyrus AC, Griffin-Blake S (2018). Prevalence of disabilities and health care access by disability status and type among adults - United States, 2016. MMWR Morb Mortal Wkly Rep.

[13] Burns N, Paterson K, Watson N (2009). An inclusive outdoors? Disabled people’s experiences of countryside leisure services. Leisure Studies.

[14] Kavanagh AM, Krnjacki L, Aitken Z, LaMontagne AD, Beer A, Baker E, Bentley R (2015). Intersections between disability, type of impairment, gender and socio-economic disadvantage in a nationally representative sample of 33,101 working-aged Australians. Disabil Health J.

[15] Accessible Canada Act (S.C. 2019, c. 10). Justice Laws Website.

[16] Accessibility standards in outdoor environments. National Park Services, USA.

[17] Jang S, Mortenson W, Hurd L, Kirby R (2019). Caught in-between: tensions experienced by community mobility scooter users. Disability Soc.

[18] Hickman K (2016). Disabled cyclists in England: imagery in policy and design. Proc Institution Civil Engineers Urban Design Planning.

[19] Ormerod M, Newton R, MacLennan H, Faruk M, Thies S, Kenney L, Howard D, Nester C (2015). Older people's experiences of using tactile paving. Proc Institution Civil Engineers Municipal Engineer.

[20] Lee H (2019). The effects of truncated dome detectable warnings on travelers negotiating curb ramps in wheelchairs. J Visual Impairment Blindness.

[21] Pour une expérience de loisir inclusive. Bulletin de l'Observatoire Québécois du Loisir.

[22] Tricco AC, Lillie E, Zarin W, O'Brien KK, Colquhoun H, Levac D, Moher D, Peters MD, Horsley T, Weeks L, Hempel S, Akl EA, Chang C, McGowan J, Stewart L, Hartling L, Aldcroft A, Wilson MG, Garritty C, Lewin S, Godfrey CM, Macdonald MT, Langlois EV, Soares-Weiser K, Moriarty J, Clifford T, Tunçalp O, Straus SE (2018). PRISMA Extension for Scoping Reviews (PRISMA-ScR): checklist and explanation. Ann Intern Med.

[23] Carpiano RM (2009). Come take a walk with me: the "go-along" interview as a novel method for studying the implications of place for health and well-being. Health Place.

[24] Hein J, Evans J, Jones P (2008). Mobile methodologies: theory, technology and practice. Geography Compass.

[25] Parent L (2016). The wheeling interview: mobile methods and disability. Mobilities.

[26] Evans J, Jones P (2011). The walking interview: methodology, mobility and place. Applied Geography.

[27] Wästerfors D (2020). Required to be creative. Everyday ways for dealing with inaccessibility. Disability Soc.

[28] Mahmood A, O’Dea E, Bigonnesse C, Labbe D, Mahal T, Qureshi M, Mortenson W (2019). Stakeholders Walkability/Wheelability Audit in Neighbourhoods (SWAN): user-led audit and photographic documentation in Canada. Disability Soc.

[29] Mahmood A, Chaudhury C, Oswald F, Konopik N (2016). Seniors Walkability Audit in Neighbourhoods (SWAN): Development of a user-led observation tool to evaluate neighbourhood design features that fosters mobility in older adults. International Association People-Environment Studies.

[30] Kaczynski AT, Stanis SA, Besenyi GM (2012). Development and testing of a community stakeholder park audit tool. Am J Prev Med.

[31] Gidlow C, van Kempen E, Smith G, Triguero-Mas M, Kruize H, Gražulevičienė R, Ellis N, Hurst G, Masterson D, Cirach M, van den Berg M, Smart W, Dėdelė A, Maas J, Nieuwenhuijsen MJ (2018). Development of the natural environment scoring tool (NEST). Urban Forestry Urban Greening.

[32] Boschmann EE, Cubbon E (2013). Sketch maps and qualitative GIS: using cartographies of individual spatial narratives in geographic research. Professional Geographer.

[33] Golledge R (1992). Place recognition and wayfinding: Making sense of space. Geoforum.

[34] Fajen B, Phillips F (2013). Spatial perception and action. Handbook of Spatial Cognition.

[35] Hochmair H, Frank A (2000). Influence of estimation errors on wayfinding-decisions in unknown street networks analyzing the least-angle strategy. Spat Cogn Comput Springer.

[36] Hund AM, Minarik JL (2006). Getting from here to there: spatial anxiety, wayfinding strategies, direction type, and wayfinding efficiency. Spatial Cognit Comput.

[37] Maus M, Lindeman D, Satariano W (2016). Wayfinding, mobility, and technology for an aging society. Community Wayfinding: Pathways to Understanding.

[38] Mortenson WB, Miller WC, Boily J, Steele B, Crawford EM, Desharnais G (2006). Overarching principles and salient findings for inclusion in guidelines for power mobility use within residential care facilities. J Rehabil Res Dev.

[39] Rushton PW, Miller WC, Lee Kirby RL, Eng JJ, Yip J (2011). Development and content validation of the Wheelchair Use Confidence Scale: a mixed-methods study. Disabil Rehabil Assist Technol.

